# SIGMA: Shear-induced gelation by microbead aggregation in tubular flow systems

**DOI:** 10.1016/j.mtbio.2026.103120

**Published:** 2026-04-13

**Authors:** Yu Ri Nam, Yeongjin Lee, Keumyeon Kim, Jeongin Seo, Jeehee Lee, Hee-Seung Lee, Haeshin Lee

**Affiliations:** a Department of Chemistry, Korea Advanced Institute of Science and Technology (KAIST), Daejeon, 34141, Republic of Korea; b R&D Center, SCL Science Inc., Seoul, 07282, Republic of Korea

**Keywords:** Gelatin microbeads, Injectable hydrogel, Shear-induced gelation, *In situ* gelation

## Abstract

Injectable hydrogels hold promise for localized drug delivery and regenerative medicine, yet their clinical translation remains limited by reliance on backbone modification or exogenous physicochemical triggers that complicate delivery. Here, we exploit an unavoidable aspect of clinical administration, shear, which is generated during injection through catheters and needles. We introduce SIGMA (Shear-Induced Gelation by Microbead Aggregation), a system composed of gelatin microbeads that remain flowable during injection but rapidly assemble into cohesive hydrogel under confined shear, without exogenous physicochemical triggers such as temperature, pH, light, or chemical crosslinkers. Gelation was tunable by varying flow path length, mixing cycles, particle sizes, or introducing air–liquid interfaces, enabling transitions from a weak dispersion (∼11.3 Pa) to robust gels with storage moduli up to ∼5 kPa and compressive strengths approaching ∼800 kPa. Circular dichroism and gel permeation chromatography revealed shear-induced intermolecular associations without evidence of chain scissions. The platform further enabled post-encapsulation drug loading with tunable release governed by shear-programmed network density. *In vitro* and *in vivo* studies confirmed cytocompatibility, biodegradability, systemic safety, and superior submucosal lift compared with saline. Together, SIGMA establishes a mechanically actuated, clinically adaptable hydrogel platform for minimally invasive surgery and localized drug delivery.

## Graphical abstract

**Figure:**
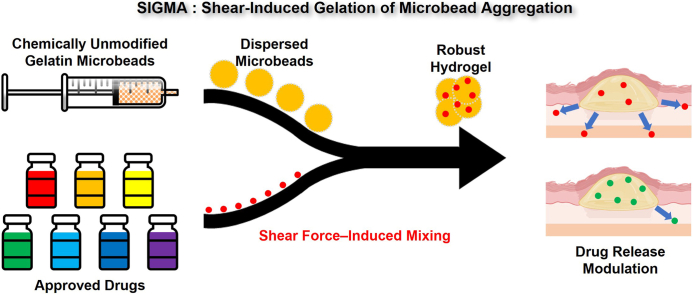

## 1 Introduction

Injectable hydrogels are valued for their ability to form three-dimensional (3D) networks *in situ*, enabling localized drug delivery, cell encapsulation, and tissue regeneration through minimally invasive procedures [[1], [2], [3]]. Over the years, a variety of gelation strategies have been engineered to trigger sol–gel transitions under physiological conditions, each designed to address distinct biomedical needs. Among them, thermoresponsive hydrogels remain the most extensively explored. Polymers such as Pluronic F127 and poly(N-isopropylacrylamide) (PNIPAM) undergo reversible sol–gel transitions near body temperature, enabling injection as liquids followed by gelation *in situ* without additional equipment [[4], [5], [6]]. Despite this conceptual simplicity, thermoresponsive systems often suffer from slow or incomplete gelation and weak mechanical strength, restricting their ability to withstand physiological stresses or maintain durable structure [[7], [8], [9]]. In addition, widely used polymers such as PNIPAM are non-degradable and pose biocompatibility concerns, further limiting their clinical applicability for long-term therapeutic use [[10], [11], [12]].

Beyond temperature-sensitive systems, other gelation strategies have been developed to broaden the design space of injectable hydrogels. pH-responsive formulations, such as chitosan, poly(acrylic acid), and their derivatives, undergo rapid sol–gel transitions in response to local acidity or alkalinity, providing the potential for site-specific gelation [[13], [14], [15]]. However, the heterogeneous and tightly regulated pH of most tissues makes it difficult to achieve reliable performance, and the need for acidic or alkaline triggers can lead to cytotoxicity and local tissue irritation, restricting their applicability in sensitive organs [[15], [16], [17]]. Photocrosslinkable systems, exemplified by gelatin methacryloyl (GelMA) and poly(ethylene glycol)diacrylate (PEGDA), offer unparalleled spatial and temporal control over other gel formation mechanisms [[18], [19], [20]]. However, they exhibit a certain level of toxicity due to unreacted photo-initiator, DNA damage due to UV lights, and suffer from limited light penetration *in vivo*, especially in deep or opaque tissues [[20], [21], [22]].

Despite these diverse approaches, the fundamental challenges of injectable hydrogels remain unresolved. Most systems rely on exogenous physicochemical triggers—such as temperature, pH, or light—to initiate gelation, which complicates clinical handling and limit reproducibility. This paradox reflects a central limitation of current injectable hydrogel systems: post-injection gelation can be readily achieved when an exogenous physicochemical stimulus accompanies the injection process; however, spontaneous *in situ* curing without external activation remains highly challenging in practice. As a result, no existing strategy simultaneously satisfies the combined requirements of injectability, mechanical robustness, biocompatibility, and clinical practicality, leaving most formulations confined to preclinical evaluation. To achieve clinical translation, injectable hydrogels must maintain injectability at the point of administration while rapidly forming mechanically stable networks *in vivo* post-injection. A promising design principle is to utilize a common environmental factor that inherently exists in versatile tubular delivery devices: shear force. Indeed, during injection through catheters, endoscopes, or other medical devices, interfacial shear is inevitably generated along the inner surfaces [[23], [24], [25]]. So far, such shear has been considered as an undesirable factor to be minimize in order to preserve material uniformity [[26], [27], [28]]. However, this conventional perspective overlooks the possibility that injection-induced shear produced by confined tubular geometries can be strategically exploited as a mechanism to drive gelation.

Previous studies have explored the concept of mechanically triggered gelation, although it has not been actively applied or optimized for practical use. Various material systems have demonstrated structural reorganization under shear. For instance, colloidal and particulate systems have shown to cluster under shear, yielding transient gel states with tunable viscoelasticity [[29], [30], [31], [32]]. Cellulose derivatives exhibit flow-sensitive sol–gel transitions, where shear influences gelation temperature and network strength [[33], [34], [35]]. Similarly, silk fibroin solutions rapidly assemble into anisotropic hydrogels under vortex or high-speed shear, demonstrating that proteins can be mechanically driven into ordered networks [[36], [37], [38]]. Collectively, these studies establish shear as a driving force for the assembling biomaterials, highlighting its potential as an alternative to conventional thermal or chemical triggers. However, previously reported shear-responsive systems exhibit critical drawbacks. Most show slow gelation kinetics, which limit their feasibility in clinical settings. In addition, these systems are generally non-tunable and mechanically weak, restricting their applicability for practical use.

Within the context of shear-induced gelation, gelatin emerges as a particularly promising biomaterial. Gelatin possesses intrinsic interfacial activity and amphiphilic character, enabling adsorption onto a wide range of interfaces, including solid–liquid, air–liquid, and oil–water boundaries. In addition, gelatin has well-established clinical safety and holds regulatory approval for various biomedical applications [[39], [40], [41], [42]]. Such interfacial activity is particularly relevant under tubular injection conditions, where hydrophobic carbon-based materials can function as oil-like interfaces that facilitate gelatin’s interfacial behavior. Nevertheless, the native physicochemical behavior of gelatin imposes a critical barrier to its application as an injectable system. Specifically, gelatin exists as a bulk gel at room temperature, hindering injection, but transitions to a sol at physiological temperature, thereby hindering the retention of structural integrity *in vivo*.

**Scheme 1:**
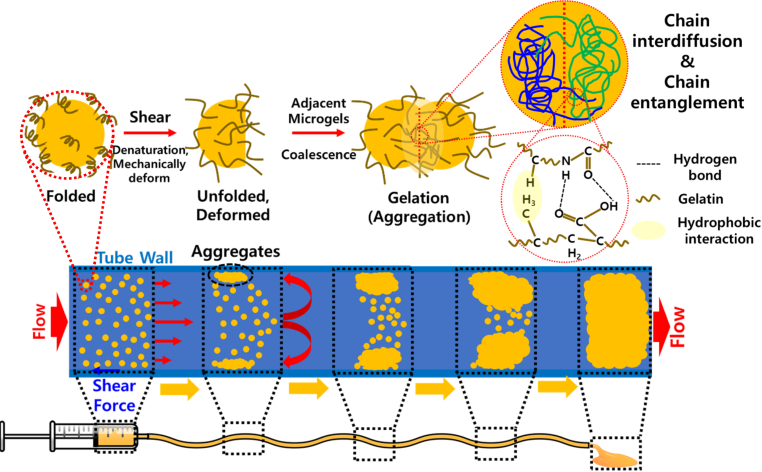
Schematic illustration of SIGMA (shear-induced gelation by microbead aggregation) in tubular flow, highlighting gelatin chain unfolding on microgel surface and bulk-level gel network formation under a clinically adaptable shear environment.

We hypothesized along two complementary lines. First, we proposed that transforming bulk gelatin into discrete microbeads would impart flowability at room temperature, thereby overcoming the immobility of gelatin in its native bulk form. In suspension, each bead maintains its internal gel structure, but collectively the system behaves as a slurry-fluid enough to be delivered through narrow catheters or syringes. Second, we anticipated that this flowable suspension, once exposed to the confined flow of clinically relevant devices, would encounter unavoidable shear that could act as an intrinsic trigger for gelation. At solid–liquid interfaces (e.g., microgel contact with syringe or catheter walls), such shear is expected to induce partial unfolding of surface gelatin chains, allowing increased lateral mobility. The anchored but mobile gelatin molecules are capable of chain-to-chain hydrogen bonding, hydrophobic association. With increasing travel distance within the confined tube, the association between gelatin microbeads progressively pronounced indicative of enhanced adhesive interactions and structural aggregation during dynamic motion. When this connectivity surpasses a critical threshold, the suspension is expected to undergo a transition from a flowable state to a cohesive hydrogel network *in situ* (Scheme 1). Together, these hypotheses provide a framework by which gelatin can be made injectable and programmed to form stable hydrogels solely through clinically unavoidable shear force, without reliance on exogenous physicochemical triggers or chemical crosslinkers.

Herein, we present a gelatin microgel system—termed SIGMA (Shear-Induced Gelation by Microbead Aggregation)—that undergoes shear-induced 3D bulk gelation using unmodified gelatin microbeads without exogenous physicochemical triggers (e.g., temperature change, pH adjustment, light irradiation, or chemical crosslinkers). This platform leverages confined flow through widely used clinical devices, where interfacial shear unfolds protein chains, allows lateral movement of the chain, and promotes robust particle–particle bonding. Gelation was systematically examined under three representative conditions to control travel length and interface area of microbeads. Passage through narrow tubing demonstrated that confined flow alone initiates network assembly. Two syringe nozzles were connected, allowing the suspension to be alternately pushed back and forth multiple times. Moreover, the introduction of air–liquid interfaces markedly accelerated gelation kinetics, leading to rapid and significant enhancement of mechanical properties. Thus, diameter of microbeads was a key morphological parameter governing interfacial area and percolation efficiency under identical shear history. By varying these interfacial parameters, SIGMA exhibited tunability from soft dispersions (∼11.3 Pa) to hydrogels with storage moduli approaching ∼5 kPa and compressive strengths near 800 kPa, without chemical modification of the gelatin backbone. To substantiate the proposed mechanism at the molecular level, shear-dependent circular dichroism (CD) and gel permeation chromatography (GPC) analyses were performed, revealing conformational activation and increased apparent molecular weight without evidence of chain scission, consistent with physically assembled, noncovalent networking. Beyond its mechanical robustness, functional assessments demonstrated the system’s applicability to clinical settings of the SIGMA. Modulation of network density by controlling travel length of microbeads enabled precise control over drug release kinetics, ranging from rapid to sustained delivery profiles. Notably, SIGMA enables a post-encapsulation strategy, in which gelatin microbeads and pharmaceutical payloads are prepared separately and subsequently encapsulated during the simple shear-injection process. *In vitro* cytocompatibility testing confirmed the absence of cytotoxicity, consistent with the intrinsic biocompatibility of gelatin. *In vivo* studies further demonstrated predictable biodegradation, favorable biocompatibility, systemic safety and complete material resorption within a short physiological timeframe. Importantly, both *ex vivo* and *in vivo* gastric models validated SIGMA as a biocompatible and efficacious submucosal injection agent (SIA), producing durable mucosal elevation and superior tissue compatibility compared with conventional approach using saline. In summary, these results establish SIGMA as a reagent-free, shear-actuated hydrogel system with significant promise for minimally invasive surgery, targeted drug delivery, and endoscopic applications such as submucosal injection agents.

## 2 Materials and methods

### 2.1 Materials

Gelatin (Type A, porcine skin) was purchased from Sammi Industry (Korea). Sorbitan oleate (nonionic surfactant) was purchased from Il Shin Wells (Korea), and paraffin oil was supplied by Molytech (Korea). Analytical-grade ethanol, acetone, and doxorubicin hydrochloride were obtained from Sigma-Aldrich (USA). All reagents were used as received without further purification unless otherwise specified.

### 2.2 Preparation of gelatin microbeads for SIGMA

Gelatin microbeads were fabricated through a water-in-oil emulsification followed by thermal gelation. Briefly, gelatin (20 wt%) was dissolved in deionized water (DW) at 60 °C for 1 h to obtain a homogeneous aqueous phase. The gelatin solution was then dispersed dropwise into an oil phase consisting of paraffin oil (7 L) and sorbitan oleate (70 mL) under constant stirring at 450 rpm and 65 °C. The dispersion was maintained for 30 min to allow uniform droplet formation. Subsequently, the emulsion was rapidly cooled to 4 °C while maintaining agitation to induce gelation of the aqueous droplets. The resulting microbeads were aged for an additional 1 h at 4 °C to ensure complete solidification, then collected by decantation. Residual oil was removed through sequential washing with acetone and ethanol. The purified microbeads were dried at ambient conditions (25 °C, 25% RH) for 24 h to remove residual solvents prior to use. To obtain three diameter populations (small/medium/large) of gelatin microbeads, the stirring speed during emulsification was varied while keeping all other parameters constant (small, 450 rpm; medium, 300 rpm; large, 200 rpm).

### 2.3 Characterization of gelatin microbeads for SIGMA

The morphology of the resulting microbeads was observed using scanning electron microscopy (SEM, S-4800; Hitachi, Japan). Particle size distribution was determined from SEM images. Three representative SEM images were analyzed, and 100 particles were measured per image (total n = 300) to calculate average diameter and size distribution. The hydrophilicity of gelatin microbeads was measured by absorption kinetics by a water droplet (20 μL).

### 2.4 Visualization of SIGMA formation during endoscopic flow

Gelatin microbeads were suspended in DW by 100 reciprocating mixing cycles between two 5 mL syringes connected by a Luer-lock adapter at 25 °C. The resulting suspension was injected into a polypropylene endoscopic tube (length = 1.8 m, inner diameter = 0.9 mm; Clear-Jet Injection Catheter, FINEMEDIX Co., Korea) at a controlled flow rate of 0.4 mL/s until completely filled. The morphological evolution of the suspension along the flow path was visually observed to confirm shear-induced gelation (SIGMA) occurring within the confined tubing.

### 2.5 Flow-distance–dependent phase transition of SIGMA in endoscopic tubing

A 20 wt% gelatin microbead suspension was prepared as described above. Immediately after mixing, 1 mL of the suspension was collected in a 2 mL vial prior to injection (0.0 m). Additional aliquots were collected after the suspension had traveled through 0.9 m segment or the full 1.8 m endoscopic tube and placed into separate vials (0.9 m and 1.8 m, respectively). Within 5 s of collection, each vial was inverted to qualitatively evaluate the flow-distance–dependent phase transition of SIGMA.

### 2.6 Optical and mechanical characterization of SIGMA along endoscopic flow pathways

To quantify turbidity changes associated with SIGMA formation, samples were collected at three positions (0.0 m, 0.9 m, and 1.8 m) along the endoscopic tube following the procedure described above. UV–Vis absorbance spectra were recorded from 400 to 800 nm using a spectrophotometer (UV-1800, Shimadzu, Japan) to evaluate optical density variations arising from shear-dependent phase transition. Mechanical properties of the same samples were evaluated using a rotational rheometer (MCR 102, Anton Paar, Austria) equipped with a 25 mm parallel-plate geometry at 25 °C. The storage modulus (G′) was measured at 1 Hz and 0.5% strain to assess shear-dependent stiffening during confined flow (n = 3).

### 2.7 Mixing-induced modulation of SIGMA storage modulus in syringe systems and size-dependent gelation tests

Gelatin microbeads (400 mg) and DW (1600 mg) were loaded into separate syringes and connected using a Luer-lock adapter. The suspension was reciprocally mixed at approximately one cycle per second for 10, 50, 100, 150, or 200 cycles. Immediately after mixing, rheological measurements were conducted using a rheometer equipped with a 25 mm parallel plate at 25 °C. The storage modulus (G′) was measured at 1 Hz and 0.5% strain (n = 3). For size-dependent gelation tests, small (12.6 ± 6.3 μm), medium (20.8 ± 15.3 μm), and large (31.9 ± 31.7 μm) microbeads were tested at the identical concentration and under the same mixing protocols (50 – 200 cycles in syringes).

### 2.8 Microstructural evolution of SIGMA under reciprocating shear

A 20 wt% gelatin microbead suspension was subjected to 10, 100, or 200 reciprocating mixing cycles. Samples were rapidly quenched in liquid nitrogen, lyophilized, and imaged using scanning electron microscopy (SEM; S-4800, Hitachi, Japan) to visualize microstructural evolution during shear-induced gelation.

### 2.9 Circular dichroism analysis and gel permeation chromatography of shear-processed GMs

CD spectroscopy was performed to evaluate shear-induced conformational changes in bulk gelatin and GMs. Five sample conditions were analyzed: (i) 20 wt% gelatin in deionized water (DW), (ii) 20 wt% gelatin in DW subjected to shear using a homogenizer at 700 rpm for 10 min, and (iii–v) 20 wt% GM suspensions in DW prepared by 10, 100, or 200 reciprocating syringe-mixing cycles, respectively. CD spectra were recorded using a circular dichroism spectrometer (J-1500, JASCO, Japan) at 25 °C. Temperature was controlled with a Peltier-type temperature controller (PFD425S/15, JASCO, Japan). Measurements were conducted from 200 to 300 nm using a quartz cuvette with a 0.1 mm path length. The scanning speed was set to 200 nm/min with a bandwidth of 1.0 nm and a data pitch of 0.5 nm. Each spectrum was averaged over three accumulations to improve signal-to-noise ratio. High-tension (HT) voltage was monitored to ensure spectral reliability. Ellipticity values were expressed in millidegrees (mdeg) and baseline-corrected against DW.

Because GMs are micron-sized particles, far-UV CD spectra may include contributions from light scattering. Therefore, systematic spectral variations as a function of shear history were interpreted as evidence of interfacial conformational activation of gelatin chains rather than as absolute quantification of bulk secondary structure content.

GPC was performed to analyze the molecular weight distribution of gelatin before and after shear processing. Two samples were prepared: (i) 20 wt% gelatin dissolved in 0.1 M NaNO_3_ and (ii) the same solution subjected to homogenization at 700 rpm for 10 min. Prior to injection, samples were diluted to 1 mg/mL with 0.1 M NaNO_3_ and filtered through a 0.22 μm syringe filter.

GPC analysis was conducted using an SB-804 HQ column (Showa Denko, Japan) maintained at 35–40 °C, equipped with a refractive index and UV detectors (214/220 nm). The mobile phase consisted of 0.1 M NaNO_3_ filtered through a 0.22 μm membrane and degassed prior to use. The flow rate was set to 0.3 mL/min, and 20 μL of each sample was injected. The run time was adjusted to ensure complete elution and baseline recovery.

Molecular weight calibration was performed using dextran standards (from Leuconostoc spp.) with nominal molecular weights of ∼40,000, ∼100,000, and 400,000–650,000 Da prepared in the mobile phase.

### 2.10 Mixing-dependent drug release behavior of SIGMA

Gelatin microbeads were combined with model payload solutions to evaluate shear-history-dependent and cargo-size-dependent release behavior of SIGMA.

For small-molecule release, gelatin microbeads (400 mg) were combined with 1600 mg of doxorubicin (DOX) solution (0.5 mg/mL) and mixed using 10, 100, or 200 syringe cycles. After mixing, 0.5 mL of each suspension was transferred into a 2 mL tube and diluted with 0.5 mL DW. At predetermined time intervals, 50 μL aliquots were withdrawn for analysis, and absorbance was measured at 485 nm (n = 3). After measurement, each aliquot was returned to the tube to preserve the total volume and maintain consistent release conditions. DOX concentrations were quantified using a pre-established calibration curve.

For macromolecular release, gelatin microbeads (200 mg) were combined with 0.8 mL of fluorescein isothiocyanate-conjugated bovine serum albumin (FITC-BSA) solution (1.0 mg/mL in PBS, pH 7.4) and mixed using 10, 100, or 200 syringe cycles. After mixing, 0.1 mL of each suspension was transferred into a 1.5 mL tube and diluted with 0.2 mL PBS. At predetermined time intervals, 0.1 mL of the supernatants were collected for analysis, and fluorescence intensity was measured at excitation/emission wavelengths of 485/520 nm (n = 3). FITC–BSA concentrations were quantified using a pre-established calibration curve.

### 2.11 Effect of Air–Liquid interfaces on SIGMA mechanics

To investigate the effect of air–liquid interfaces during flow, a 20 wt% gelatin microbead suspension (0.6 mL) was passed through a 0.9 m endoscopic tube under four distinct interface conditions. For the baseline (Sample 0), the trailing 0.6 mL fraction was collected after injecting the suspension without air exposure. Sample 1 consisted of the leading 0.6 mL fraction from the same injection to examine front-end shear effects. To introduce controlled air–liquid interfaces, Sample 2 was prepared by sequentially injecting 0.3 mL of suspension, 0.3 mL of air, and an additional 0.3 mL of suspension, resulting in a total collected volume of 0.6 mL. Sample 3 was designed to maximize interfacial contact by alternately injecting 0.15 mL of suspension and 0.15 mL of air four times. All collected samples were analyzed using rotational rheometer equipped with a 25 mm parallel plate geometry at 25 °C. The storage modulus (G′) was measured at an oscillatory frequency of 1 Hz and a strain amplitude of 0.5% (n = 3).

### 2.12 Air-fraction–dependent mechanical transition of SIGMA during syringe mixing

Gelatin microbeads (400 mg) were loaded into one syringe, while another contained 1.6 mL of DW with 0, 0.8, 1.6, or 3.2 mL of air. The syringes were connected and mixed reciprocally at ∼1 cycle/s for 100 cycles. After resting for 1 min to allow air–liquid phase separation, the air phase was removed, and the liquid phase was analyzed rheologically (25 °C, 25 mm plate, 1 Hz, 0.5% strain).

### 2.13 Bulk compression testing of SIGMA

SIGMA samples (20 wt% gelatin microbeads) were prepared under four conditions: 50 cycles, 100 cycles, 100 cycles with 100% additional air (V/V), and 200 cycles with 100% additional air. The samples were cast in cylindrical molds (1 cm^2^ cross-sectional area, 1 cm height) and subjected to uniaxial compression using a universal testing machine (EZ-LX, Shimadzu, Japan) at 0.1 mm/s until 80% strain.

### 2.14 *In vitro* cytotoxicity assessment of SIGMA using L929 cells

*In vitro* cytotoxicity of SIGMA was evaluated using mouse fibroblast L929 cells. Cells were cultured in Dulbecco’s Modified Eagle Medium (DMEM) supplemented with 10% fetal bovine serum (FBS) and 1% penicillin–streptomycin under standard conditions (37 °C, 5% CO_2_). For cytotoxicity evaluation, cells were seeded in 24-well plates at an appropriate density and allowed to attach for 24 h. The culture medium was then replaced with fresh medium containing one of the following conditions: (i) negative control (DMEM), (ii) positive control (Latex), (iii) conventional gelatin (10 mg/mL), or (iv) SIGMA (10 mg/mL). Cells were incubated under these conditions for 24 h prior to analysis. Cell viability was qualitatively assessed using a LIVE/DEAD staining assay. After 24 h incubation, cells were washed with phosphate-buffered saline (PBS) and stained according to the manufacturer’s protocol. Fluorescence images were obtained using a fluorescence microscope (Nikon Eclipse Ti, Nikon, Japan). Live cells were stained green, and dead cells were stained red. Quantitative cell viability was evaluated using a Cell Counting Kit-8 (CCK-8) assay. After 24 h exposure to each condition, CCK-8 reagent was added to each well and incubated for the recommended duration at 37 °C. Absorbance was measured at 450 nm using a microplate reader (Varioskan Flash, Thermo Fisher Scientific, USA). Cell viability was expressed as a percentage relative to the negative control group. All experiments were performed in triplicate (n = 3).

### 2.15 *In vivo* biocompatibility of SIGMA in a rabbit intradermal model

Intradermal biocompatibility was evaluated in two New Zealand White rabbits following ISO 10993-10 and ISO 10993-12 guidelines (Knotus Co. Ltd., South Korea; IACUC No. 22-KE-0062). Dorsal fur was shaved prior to injection. Gelatin microbeads were extracted in saline or cottonseed oil (0.2 g/mL) for 72 h at 37 °C. Each rabbit received 0.2 mL intradermal injections of saline, cottonseed oil, gelatin extract in saline, and gelatin extract in cottonseed oil. Injection sites were scored for erythema (ER) and edema (OE) at 24, 48, and 72 h following ISO 10993-10 criteria.

### 2.16 *In vivo* biodegradation and safety evaluation of SIGMA in rat subcutaneous injection model

The *in vivo* biodegradation and safety of gelatin microbeads (GMs) were evaluated in male Sprague–Dawley rats (≈300 g, 9 weeks old) according to ISO 10993-1, −6, and −12 guidelines. 20 wt% gelatin microbead suspension prepared by 10, 100, or 200 reciprocating syringe-mixing cycles were injected subcutaneously (0.3 mL per site) into separate dorsal sites. A non-injected site served as a local control. Anesthesia was induced by intramuscular administration of Zoletil 50 (tiletamine and zolazepam, 33.3 mg/kg, Virbac, France) and Rompun (xylazine, 7.8 mg/kg, Bayer, Germany). Animals were sacrificed at 0 h, 6 h, 24 h, 72 h, 1 week, and 2 weeks post-injection via CO_2_ asphyxiation. At each time point, injection-site tissues were excised. For residual mass analysis, explanted tissues (n = 5 per time point) were frozen, lyophilized, and weighed. Residual mass (%) was calculated relative to samples excised immediately after injection (0 h reference). For histological evaluation, injection-site tissues at each time points (including control and GM-injected sites) were fixed in 10% neutral buffered formalin, processed, and stained with hematoxylin and eosin (H&E) to assess local inflammatory responses. For systemic toxicity assessment, a separate group of rats received subcutaneous injections of either GMs (three sites, 0.3 mL per site) or phosphate-buffered saline (PBS, three sites, 0.3 mL per site). At 2 weeks post-injection, blood samples were collected from the inferior vena cava (n = 6 per group). Serum biochemical parameters, including alanine aminotransferase (ALT), aspartate aminotransferase (AST), blood urea nitrogen (BUN), and creatinine were analyzed using an automated clinical chemistry analyzer. At 2 weeks post-injection, major organs (heart, liver, spleen, lung, and kidney) were harvested, fixed in 10% neutral buffered formalin, sectioned, and subjected to H&E staining for histopathological evaluations. All animal procedures were approved by the Institutional Animal Care and Use Committee of KAIST (Approval No. KA2024-175) and complied with the ethical guidelines of the Korean Ministry of Health and Welfare.

### 2.17 *Ex vivo* evaluation of submucosal elevation stability of SIGMA under varying mixing conditions

Submucosal elevation stability of SIGMA was assessed using porcine gastric tissue model maintained at 37 °C and 80% relative humidity. Each sample (1 mL) of SIGMA prepared by (1) 100 mixing cycles, (2) 100 cycles with an additional air volume equivalent to 100% DW, (3) 200 cycles with 100% added air, and (4) DW (control) were injected into the submucosa using an 18G needle. Elevation height and morphology were monitored over time, and morphological changes was evaluated by comparing stability and persistence among groups.

### 2.18 Porcine ESD model for *In vivo* evaluation of SIGMA

The biocompatibility and handling performance of SIGMA were assessed in a porcine endoscopic submucosal dissection (ESD) model. The study was approved by the Animal Experiment Committee (IACUC No. DGMIF-20100605-01) and conducted at the Experimental Animal Center of the Daegu Gyeongbuk Advanced Medical Industry Promotion Foundation (DGMIF), Daegu, South Korea. One female pig (CV Micropig®, Apures, Korea) was fasted for 24 h prior to the procedure. The anesthesia was induced with intramuscular Zoletil 50 (5 mg/kg) and Rompun (2 mg/kg). After securing intravenous access, the animal was intubated with a 6–7 Fr endotracheal tube, and general anesthesia was maintained with 1 L/min O_2_, 2–3 vol% isoflurane, 10–15 mL/kg tidal volume, and 20–25 breaths/min at 60% inspired O_2_. All procedures were performed by an experienced gastroenterologist. Submucosal injections (2 mL each) of saline or 20 wt% SIGMA (100 mixing cycles) were administered through a 1.8 m endoscopic tube. After 2 h observation, ESD was performed at the injection sites using a single-channel endoscope (GIF-XQ260; Olympus, Japan) with a light source (EVIS CLV260SL) and video processor (EVIS LUCERA CV-260SL). Dissection was carried out using an insulated-tip knife (Olympus, Japan) in endo-cut mode following standard techniques. Resected tissues were fixed in 10% neutral buffered formalin and processed for H&E staining. Histological evaluation was performed by a board-certified gastrointestinal pathologist.

### 2.19 Effects of surface hydrophilicity on shear-induced gelation of SIGMA

To investigate the effect of surface wettability on shear-induced gelation, hydrophobic GMs were prepared under vacuum (10^−3^ Torr) at 25 °C for 24 h. GMs dried in ambient conditions (25 °C, 25% RH) provided hydrophilic surfaces. The hydrophobic and hydrophilic GMs were each mixed with deionized water (DW) using a syringe-to-syringe mixing method as described in Section 2.7. Two hydration conditions were evaluated: (i) immediate mixing (0 min resting) in which GMs and DW were directly subjected to 100 reciprocating mixing cycles, and (ii) pre-hydration for 60 min prior to mixing, in which GMs and DW were loaded into one syringe, allowed to rest for 60 min at room temperature, and then mixed for 100 cycles. The storage modulus (G′) of each sample was measured using a rotational rheometer (MCR 102, Anton Paar, Austria) equipped with a 25 mm parallel-plate geometry at 25 °C, at 1 Hz and 0.5% strain (n = 3).

### 2.20 Statistical analysis

Data are presented as mean ± standard deviation (SD). Statistical significance between groups was determined using Student’s t-test, with a p-value <0.05 considered statistically significant.

## 3 Results and discussion

### 3.1 Shear-induced phase transition of gelatin microbeads under endoscopic tubing

**Fig. 1:**
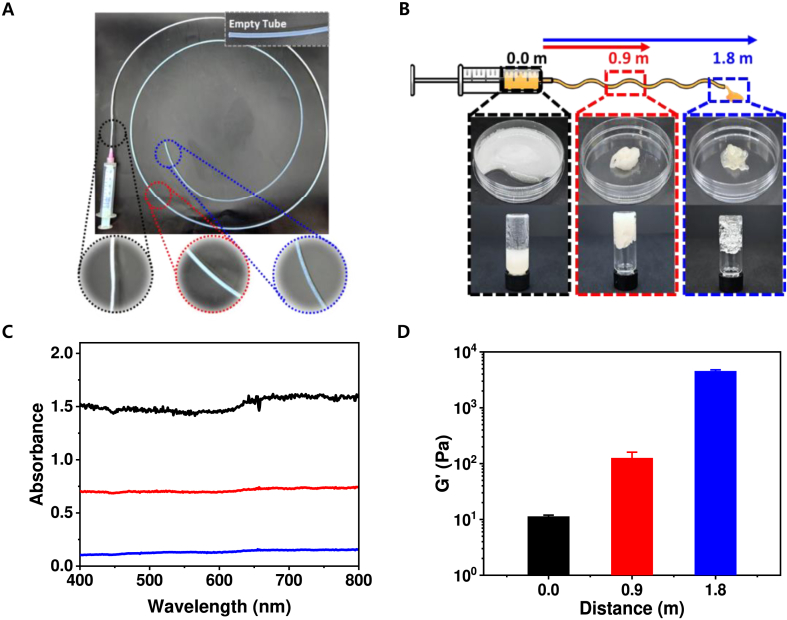
Shear-dependent gelation of gelatin microbeads in tubular flow. (A) Samples were injected through a single tube with collection points at different path lengths (black, 0.0 m; red, 0.9 m; blue, 1.8 m). (B) Photographs of 20 wt% gelatin microbead suspensions immediately collected during travel (upper), and the materials were transferred to vials which were upside down to show gelation in a qualitative manner (lower). (C) Turbidity of collected samples measured by UV–Vis spectrophotometer (black, 0.0 m; red, 0.9 m; blue, 1.8 m). (D) Storage modulus (G′) of collected samples determined by a rheometer (n = 3, mean ± SD). (For interpretation of the references to color in this figure legend, the reader is referred to the Web version of this article.)

To verify whether confined flow can induce gelation through interfacial shear, a 20 wt% gelatin microbead suspension was injected through an endoscopic tube. Samples examined at defined positions along the flow path (0.0, 0.9, and 1.8 m) revealed a clear visual transition along the tubing (Fig. 1A). At the inlet (0.0 m, black circle), the suspension appeared opaque and white, consistent with light scattering by freely dispersed gelatin microbeads. After traveling 0.9 m (red circle), the opacity decreased, and the disappeared blue color by the bead scattering began to appear, suggesting partial coalescence of neighboring beads. At 1.8 m (blue circle), the suspension became almost transparent, indicating extensive association and the formation of a continuous network. The morphology of the extruded samples supported these visual observations (Fig. 1B). The 0.0 m sample remained a flowable slurry (**left**), whereas 0.9 m sample showed a self-supporting gel lump (**middle**), and the 1.8 m sample produced a cohesive, elastic plug that retained its shape even after vial inversion (**right**). These results demonstrate a progressive shear-induced transition from discrete microbeads to a cohesive hydrogel network. Optical analysis further confirmed this transition. UV–Vis spectra exhibited a progressive decrease in light scattering across 400–800 nm with increasing travel distance (Fig. 1C), indicating reduced turbidity as microbead particles coalesced into a bulk network. Rheological measurements revealed a corresponding increase in storage modulus (G′), rising from approximately 11 Pa at 0.0 m to 126 Pa at 0.9 m and 4.5 kPa at 1.8 m. This travel length-dependent stiffening quantitatively confirms that interfacial shear within confined flow drives robust network formation (Fig. 1D). Collectively, these findings establish a practical framework in which catheter geometry can be used to program gelation at the target site, enabling minimally invasive hydrogel delivery. Because the injection parameters used in this study fall within clinically reported ESD ranges, the system supports reproducible performance under routine clinical conditions [[43], [44], [45]].

### 3.2 Shear-induced gelation through reciprocating syringe mixing and its impact on drug release

**Fig. 2:**
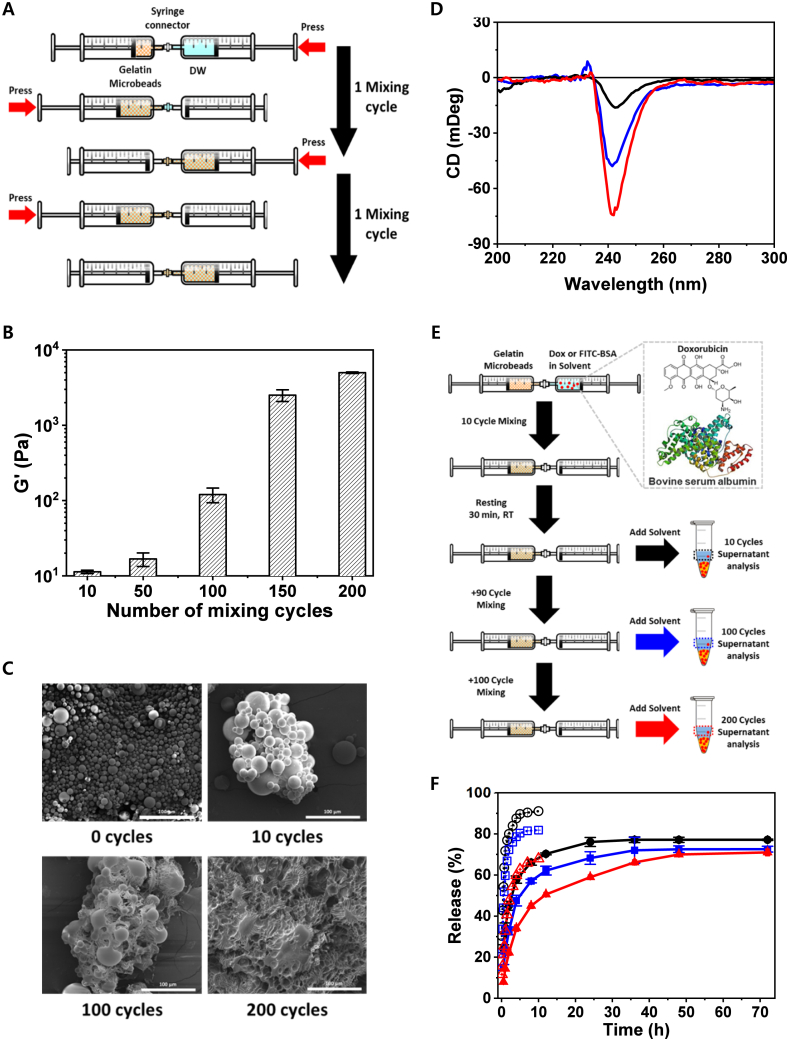
Shear-induced gelation of gelatin microbeads under repeated syringe mixing. (A) Schematic illustration of the syringe-to-syringe mixing system designed to generate controlled cyclic shear. (B) Storage modulus (G′) of SIGMA as a function of mixing cycles (n = 3, mean ± SD). (C) SEM images of microbeads after 0 cycles (top left), 10 cycles (top right), 100 cycles (bottom left), and 200 cycles (bottom right), (scale bar = 100 μm). (D) CD spectra of SIGMA prepared by 10 (Black), 100 (Blue), and 200 (Red) mixing cycles. (E) An experimental scheme of DOX and FITC-BSA loading into SIGMA and subsequent release analysis under varying the number of mixing cycles. (F) DOX and FITC-BSA release profiles prepared with different mixing cycles (dot lines, DOX; solid lines, FITC-BSA; black circle, 10; blue rectangle, 100; red triangle, 200 cycles; n = 3, mean ± SD). (For interpretation of the references to color in this figure legend, the reader is referred to the Web version of this article.)

In clinical practice, biomaterials are subject to experience repeatedly shear stress as they pass through narrow catheters or endoscopic needles. To reproduce this effect under controlled laboratory conditions, we established a benchtop model using reciprocating syringe mixing. Two Luer-locked syringes—one containing a 20 wt% gelatin microbead suspension and the other containing deionized water (DW)—were connected. One mixing cycle was defined as transferring the contents completely from one syringe to the other and back again, ensuring reproducible exposure to confined shear along the syringe walls (Fig. 2A).

Prior to shear experiments, morphological properties of the fabricated GMs were characterized. GM exhibited smooth, non-porous spheres with a relatively narrow size distribution (mean diameter 12.6 ± 6.3 μm, PDI 0.25; n = 300) (Fig. S1A, B left). Surface wettability was evaluated using a contact-angle analyzer by monitoring water-droplet absorption kinetics (20 μL). Water droplets were completely absorbed within ∼1 s, confirming a highly hydrophilic surface state (Fig. S2A).

Rheological measurements revealed a clear shear-history-dependent increase in storage modulus (G′). After 10 cycles, G′ was ∼11 Pa, indicating a weakly structured dispersion. At 100 cycles, G′ increased to ∼120 Pa, while 200 cycles yielded ∼4994 Pa, comparable to values observed in endoscopic tube experiments (Fig. 1D). This trend indicates that cumulative shear transforms a flowable slurry into a mechanically cohesive hydrogel network (Fig. 2B).

SEM imaging corroborated this structural evolution. At 0 cycle, discrete spherical microbeads with well-defined boundaries were observed, confirming the absence of spontaneous interparticle fusion. After 10 cycles, loosely connected particles appeared. At 100 cycles, interfacial necking and partial fusion were clearly observed. By 200 cycles, a continuous porous scaffold had formed (Fig. 2C). Importantly, bead boundary remained identifiable, indicating that macroscopic modulus enhancement arises from progressive interfacial consolidation. Although the reduced boundary contrast at higher mixing cycles may suggest particle disruption, no widespread fracture or fine particulate debris was observed. Instead, the structural transition is more consistent with increased bead–bead contact and interfacial integration, indicating that the modulus enhancement arises from progressive junction formation between beads rather than particle fragmentation by shear force.

To probe structural evolution at the molecular level, CD spectroscopy was performed. Native gelatin exhibited a negative ellipticity peak at 239.5 nm, whereas shear-experienced gelatin showed a red-shift of this peak to approximately 243.5 nm with increased negative magnitude, which is characteristic of disrupted helical domains and a more unstructured gelatin conformation [46,47] (Fig. S3A). In GM suspensions, ellipticity increased progressively with cycle number (10, 100, 200 cycles: −16.18, −47.88, and −74.42 mdeg, respectively), indicating cumulative activation of surface-associated chains under confined shear (Fig. 2D). GPC further revealed an increase in apparent molecular weight from ∼57 kDa to ∼125 kDa, accompanied by the emergence of high-molecular-weight species in the multi-megadalton range (Fig. S3B). Notably, no low-molecular-weight fragments were detected, excluding the possibility of shear force-induced chain scission. Instead, these results indicate that shear-activated surface gelatin chains undergo intermolecular association, consistent with chain-to-chain physical aggregation rather than molecular degradation [48,49]. Together, these data support a mechanism in which confined shear promotes conformational activation and intermolecular association of gelatin chains at bead interfaces, leading to progressive network consolidation without chemical crosslinkers.

Because shear-induced gelation occurs at bead interfaces, we next examined whether particle geometry influences network formation efficiency. Three GM populations with distinct mean diameters—small (12.6 ± 6.3 μm, PDI 0.25), medium (20.8 ± 15.3 μm, PDI 0.54), and large (31.9 ± 31.7 μm, PDI 0.99)—were subjected to identical syringe-mixing (50–200 cycles) at the same concentration (20 wt%) (n = 3) (Fig. S1B). Across all conditions, G′ consistently followed the order small (2514.6 Pa) > medium (934.7 Pa) > large (365.1 Pa) at 150 cycles (Fig. S1C). Smaller microbeads provide large interfacial area per unit volume and increased frequent bead-to-bead contacts under identical shear history, thereby accelerating percolation and mechanical reinforcement [[29], [30], [31]]. These findings demonstrate that shear-induced gelation in SIGMA is governed by particle sizes and interfacial design rather than stochastic aggregation.

Surface wettability of GMs influenced early-stage gelation kinetics. Ambient-dried GMs exhibited hydrophilic surfaces showing rapid droplet absorption (∼1 s), whereas vacuum-dried GMs showed delayed absorption (∼6 s), indicating rather hydrophobic surfaces. Ambient-dried GMs displayed faster modulus development under identical shear conditions because of rapid hydration (Fig. S2B). However, after sufficient pre-hydration, both systems converged to comparable G′ values, indicating that hydration modulates gelation kinetics while confined shear remains the dominant trigger of network formation.

Because network density governs diffusional transport [50], we evaluated shear-history-dependent drug release using both small-molecule (DOX) and macromolecular (FITC-BSA) payloads. DOX- and FITC-BSA-loaded GMs prepared using 10, 100, or 200 cycles were overlaid with DW or PBS (Fig. 2E). Release kinetics correlated with gel cohesion. For DOX, after 10 cycles, the drug was rapidly released (∼72% at 1 h; ∼91% at 10 h). The 100-cycle sample showed intermediate release (∼60% at 1 h; ∼82% at 10 h). The 200-cycle sample exhibited sustained release (∼59% at 4 h; ∼68% at 10 h). For FITC-BSA, release occurred over a significantly extended timescale, with ∼58% at 4 h and ∼77% at 36 h for 10 cycles, ∼62% at 12 h and ∼73% at 48 h for 100 cycles, and ∼59% at 24 h and ∼71% at 72 h for 200 cycles (Fig. 2F).

Notably, while both payloads exhibited consistent shear-history-dependent trends, the extent and timescale of modulation differed depending on payload size. The markedly delayed release of FITC-BSA (∼66.7 kDa) compared to DOX (∼0.5 kDa) indicates that transport is governed by the effective mesh size of the inter-bead network, rather than by simple dissolution, thereby establishing release in SIGMA as a shear-tunable, network-mediated transport phenomenon. Thus, shear history directly programs both stiffness and transport behavior in SIGMA.

Importantly, post-encapsulation during shear-induced assembly—without modifying the base material—may provide regulatory and translational flexibility for clinical applications. Together, Fig. 2 shows that mechanical history alone—encoded as discrete syringe-mixing cycles across a simple miscible interface—controls both mechanical (modulus) and transport (drug release) properties of SIGMA.

### 3.3 Effect of air–water interfaces on shear-induced gelation and mechanical reinforcement

**Fig. 3:**
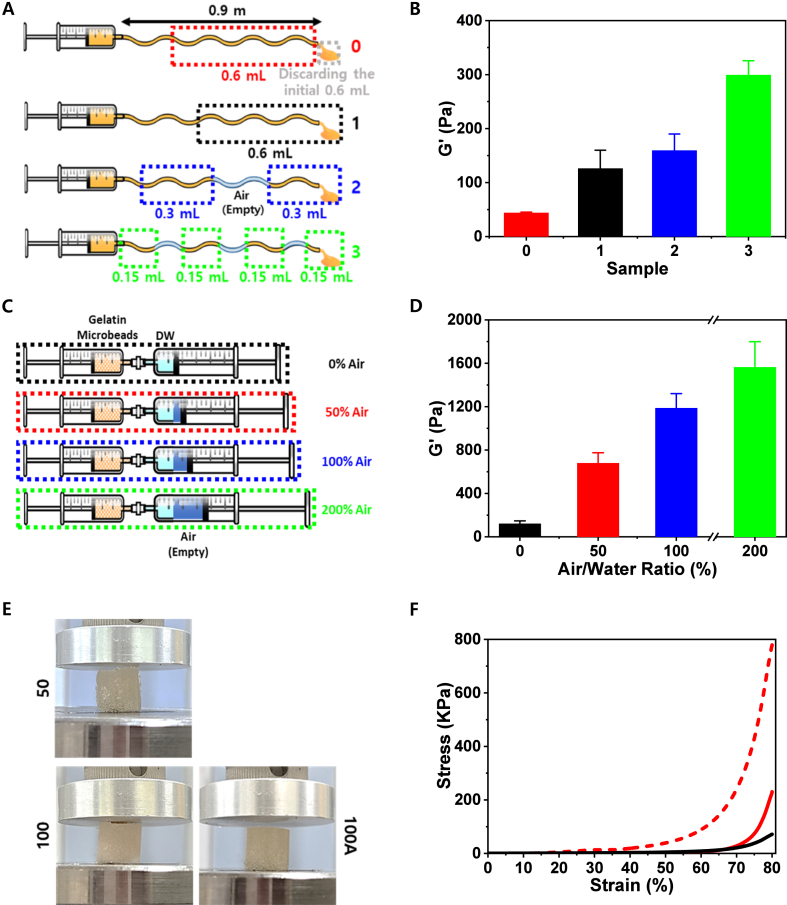
Effect of air–water interfaces on SIGMA. (A) A schematic illustration of the endoscopic tube setup designed to evaluate the influence of air–water interfaces during injection. (B) Storage modulus (G′) of gels collected from (A) after passing through 0 (red), 1 (black), 2 (blue), or 3 (green) interfaces (n = 3, mean ± SD). (C) A schematic illustration of shear-induced hydrogel formulations by increasing air volume relative to water one (0%, 50%, 100%, or 200% air) (light blue for DW and dark blue for air). (D) Storage modulus (G′) of gels collected from (C), expressed as a function of air fraction (0%, black; 50%, red; 100%, blue; 200%, green; n = 3, mean ± SD). (E) Representative images from uniaxial compression tests for gels prepared by 50 (top left) and 100 (bottom left) mixing cycles without air, and by 100 cycles with added air (100A, bottom right). (F) Corresponding stress–strain curves from the compression tests shown in (E) (50 for black, 100 for red, and 100 cycles with air for red dash). (For interpretation of the references to color in this figure legend, the reader is referred to the Web version of this article.)

The preceding results demonstrated that shear-induced gelation occurs primarily at solid (the inner walls of tube or syringe surfaces)–liquid interfaces. In addition to these solid boundaries, air–water interfaces can also serve as potent mechanical triggers for gelation. The discontinuity at the liquid–air boundary imposes localized stresses that unfold gelatin chains, resulting in interparticle bonding. To test this effect, controlled air pockets were introduced into gelatin microbead suspensions, and their influence on gelation was systematically assessed. Gelatin microbead suspensions were passed through a 0.9 m endoscopic tube under four conditions (Fig. 3A): without air (Sample 0, red), with a single leading-edge interface (Sample 1, black), and with two (Sample 2, blue) or three (Sample 3, green) inserted air pockets. Rheological analysis revealed a clear dependence of gel stiffness on the number of air-water interfaces. G′ increased from ∼44 Pa with no interface to ∼126 Pa with one, ∼159 Pa with two, and ∼299 Pa with three (Fig. 3B), supporting the hypothesis that increased exposure to air–water boundaries accelerate protein unfolding and network formation. This principle was further examined using the syringe-mixing system, where transient air–water interfaces could be introduced in a controlled manner. Gelatin microbead were mixed with 0%, 50%, 100%, or 200% air (relative to water volume) and subjected to 100 mixing cycles (Fig. 3C). Consistent with the endoscopic tube experiments, the inclusion of air significantly enhanced gel stiffness (Fig. 3D). The storage modulus (G′) increased progressively with higher air fractions, rising from approximately 120 Pa (0% air) to ∼678 Pa (50%), ∼1186 Pa (100%), and ∼1563 Pa (200%). Uniaxial compression tests further demonstrated the macroscopic reinforcement conferred by air interfaces (Fig. 3E and F). Gels mixed for 50 cycles without air were weak and poorly shaped (∼72 kPa) (Fig. 3E, top left; Fig. 3F, black). After 100 cycles without air, compressive strength improved to ∼230 kPa with more uniform structure (Fig. 3E, bottom left; Fig. 3F, red). Incorporating air dramatically enhanced mechanical performance: 100-cycle air-mixed samples reached ∼779 kPa and exhibited superior homogeneity (Fig. 3E, bottom right; Fig. 3F, red dash). Together, these findings confirm that air–water interfaces act as amplifiers of shear-induced gelation. Increasing interface areas—through segmented injection or air-assisted mixing—accelerate network formation and enhances bulk strength, achieving compressive moduli up to ∼779 kPa. Importantly, this reinforcement occurs without heat, light, or chemical crosslinkers, underscoring air–water interfaces as a simple, reagent-free, and clinically compatible trigger for programming mechanical properties of SIGMA.

### 3.4 Cytotoxicity of SIGMA

**Fig. 4:**
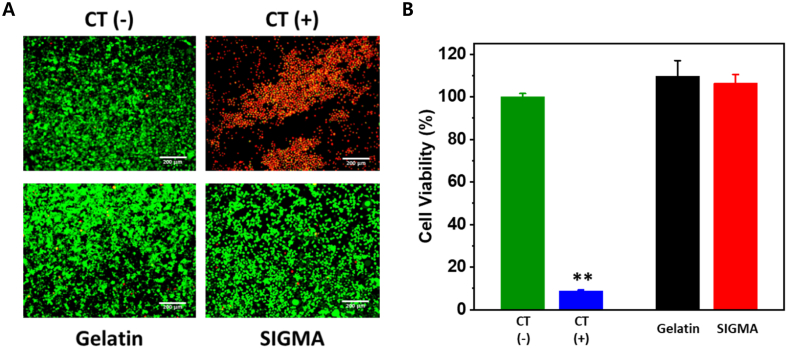
Cytotoxicity of gelatin hydrogel prepared by SIGMA. (A) LIVE/DEAD staining assay for assessing cytotoxicity of conventional gelatin and SIGMA on L929 cells after 24-h incubation under different conditions: negative control (DMEM), positive control (Latex), and medium containing 10 mg/mL of conventional gelatin or SIGMA (green: live, red: dead, scale bar: 200 μm). (B) Quantitative analysis of cell viability (%) based on the cell counting kit-8 (CCK-8) assay following 24-h incubation with negative control (Green, DMEM), positive control (Blue, Latex), conventional gelatin (Black, 10 mg/mL), and SIGMA (Red, 10 mg/mL) (n = 3, mean ± SD, **p < 0.005). (For interpretation of the references to color in this figure legend, the reader is referred to the Web version of this article.)

To further validate the biocompatibility of SIGMA at the cellular level, we performed an *in vitro* cytotoxicity assessment using L929 mouse fibroblasts, a standard cell line recommended for biological evaluation of medical materials under ISO 10993-5. Cells were incubated for 24 h in media containing 10 mg/mL of conventional gelatin or SIGMA, alongside negative (DMEM) and positive (Latex) controls. LIVE/DEAD staining revealed a high proportion of viable (green) cells in both the gelatin- and SIGMA-treated groups, comparable to the negative control, whereas the Latex group exhibited a pronounced increase in dead (red) cells (Fig. 4A). These qualitative observations were consistent with quantitative CCK-8 analysis. Relative to the negative control (100%), the gelatin-treated group showed 109.7% cell viability, and the SIGMA-treated group showed 106.4% viability (n = 3, mean ± SD), both significantly higher than the positive control (**p < 0.005) (Fig. 4B). Importantly, these values are well above the ISO 10993-5 cytotoxicity threshold of 70% viability, indicating that neither conventional gelatin nor SIGMA induces cytotoxicity under the tested conditions. Collectively, these *in vitro* results demonstrate that shear-induced gelation does not introduce adverse cellular effects and that SIGMA maintains cytocompatibility comparable to native gelatin. These findings are consistent with the subsequent *in vivo* safety evaluations and further support the translational potential of SIGMA as a clinically adaptable injectable hydrogel system.

### 3.5 *In vivo* biocompatibility and biodegradation of SIGMA

**Fig. 5:**
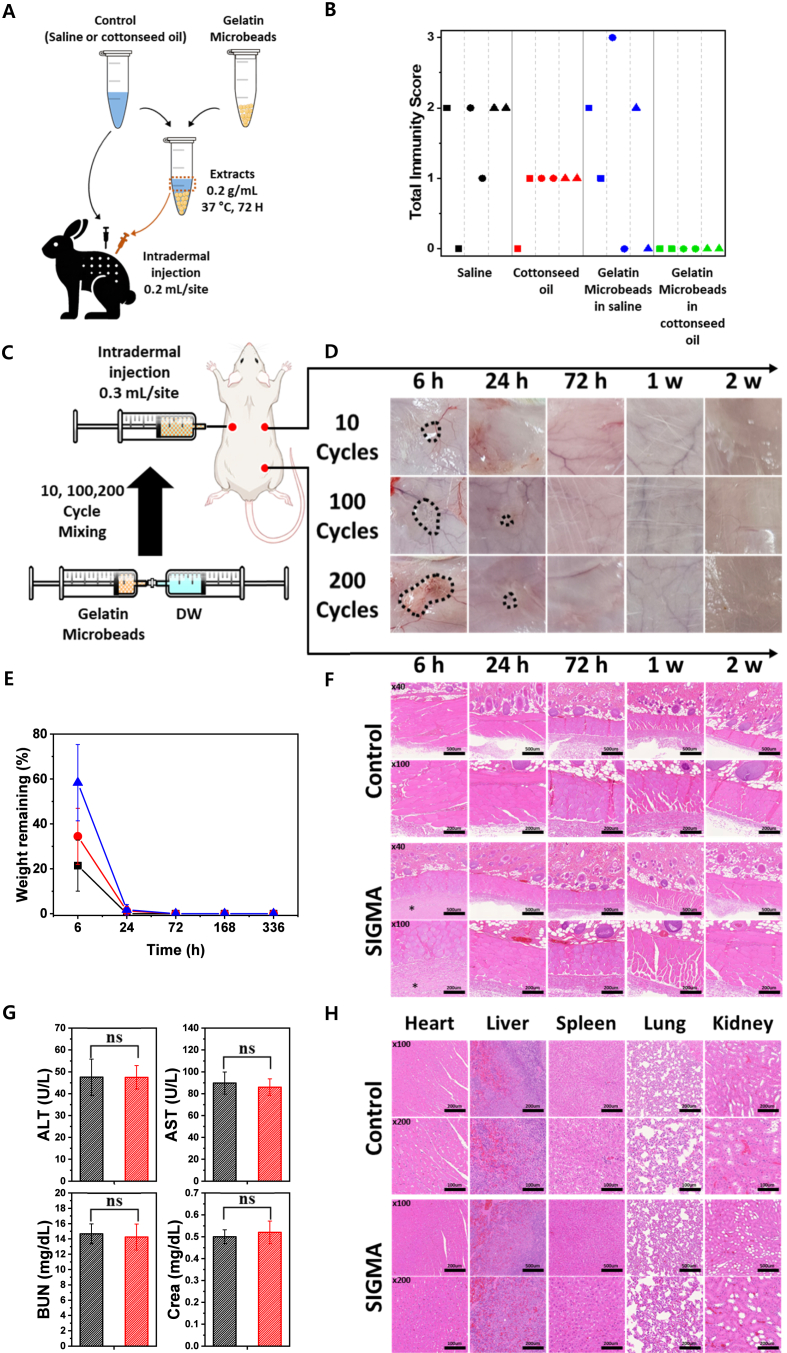
*In vivo* reactivity and biocompatibility tests of SIGMA. (A) Schematic illustration of the intradermal reactivity assay performed in New Zealand White rabbits. (B) Combined erythema and edema immunity scores at 24 (square), 48 (circle), and 72 h (triangle) after injection of saline (black), cottonseed oil (red), gelatin microbeads in saline (blue), and gelatin microbeads in cottonseed oil (green). (C) Schematic of the *in vivo* biodegradation assay conducted in a rat dorsal skin model. (D) Representative photographs showing *in vivo* degradation of SIGMA prepared with 10, 100, and 200 mixing cycles at 6 h, 24 h, 72 h, 1 week, and 2 weeks after subcutaneous injection in SD rats. (E) Quantitative degradation kinetics of subcutaneously injected SIGMA prepared by 10 (black solid line), 100 (red solid line), and 200 (blue solid line) mixing cycles (n = 6, mean ± SD). (F) Representative H&E-stained histological sections of subcutaneous tissues at 6 h, 24 h, 72 h, 1 week, and 2 weeks after injection of control (PBS) and SIGMA prepared with 100 mixing cycles. (G) Blood chemistry analysis at 2 weeks post-injection comparing PBS (Black) and SIGMA prepared with 100 mixing cycles (Red) (ALT, AST, BUN, and creatinine; n = 6, mean ± SD). (H) Representative H&E-stained sections of major organs (heart, liver, spleen, lung, and kidney) collected 2 weeks after injection of PBS or SIGMA prepared with100 mixing cycles. (For interpretation of the references to color in this figure legend, the reader is referred to the Web version of this article.)

We next evaluated the *in vivo* safety profiles of SIGMA, focusing on two essential requirements for clinical translation: local biocompatibility and biodegradation. To evaluate potential inflammatory reactivity, an intradermal irritation assay was performed in New Zealand White rabbits. The extract solution from gelatin microbeads were prepared in accordance with the ISO 10993 guidelines and subsequently injected (0.2 mL per site) into the dorsal skin, with saline and cottonseed oil serving as controls. Erythema and edema were scored at 24, 48, and 72 h and summed to obtain overall reactivity scores (Fig. 5A). According to ISO 10993 criteria, an average immunity scores do not exceed one point above the saline control is considered to be non-reactive. Across all time points, the total immunity score of gelatin microbead groups remained comparable to or lower than those of the controls, indicating minimal inflammatory response and excellent local tolerability (Fig. 5B).

To examine *in vivo* degradation under subcutaneous implantation conditions using Sprague–Dawley rats (0.3 mL per site; n = 6). SIGMA hydrogels prepared with different shear histories (10, 100, and 200 mixing cycles) were injected subcutaneously, and residual material mass was quantified at predetermined time points (Fig. 5C). At 6 h post-injection, degradation exhibited clear shear-history dependence. Residual mass was 21.4% ± 11.3 (10 cycles), 34.4% ± 12.5 (100 cycles), and 58.3% ± 17.0 (200 cycles), demonstrating that increased shear history—and thus higher network density—conferred enhanced early retention (Fig. 5D and E). Despite this initial difference, all formulations underwent rapid bio-resorption thereafter. By 24 h, the 10-cycle group was fully degraded, while only trace residues were detected in the 100- and 200-cycle groups (1.2% ± 2.1 and 1.7% ± 2.3, respectively). No detectable material remained at 72 h, 1 week, or 2 weeks, confirming complete biodegradation within a short physiological timeframe. Notably, even under an increased injection volume (1 mL per site), no residual material was detected at 8 weeks post-implantation, indicating complete long-term resorption under higher load conditions (Fig. S4). Histological analysis supported these findings (Fig. 5F and Fig. S5). At 6 h, the injected material was identifiable without indications of excessive inflammatory cell infiltration, necrosis, or abscess formation. At later time points (1 and 2 weeks), chronic inflammation, fibrotic capsule formation, and abnormal tissue remodeling were not observed. Tissue architecture was comparable to PBS controls, indicating resolution without pathological observations.

Systemic safety was evaluated at 2 weeks post-injection using serum biochemical markers to evaluate potential liver and kidney toxicity (Fig. 5G). Alanine aminotransferase (ALT) and aspartate aminotransferase (AST) were measured as indicators of hepatic function, while blood urea nitrogen (BUN) and creatinine were used to assess renal function. No statistically significant differences were observed between PBS and SIGMA groups in ALT, AST, BUN, or creatinine levels (n = 6, all ns). Histopathological analysis of major organs—including the heart, liver, spleen, lung, and kidney—was also performed to evaluate organ-level safety, revealing no structural abnormalities or inflammatory lesions compared with PBS controls (Fig. 5H).

In summary, these results demonstrate that the shear-activated gelation platform provides mechanically programmable early stability while remaining fully biodegradable and systemically safe. Regardless of controlling network density by shear history, all formulations ultimately undergo complete resorption without chronic tissue response. These characteristics support the suitability of SIGMA as a clinically adaptable, shear-triggered injectable scaffold platform.

### 3.6 *Ex vivo* and *In vivo* evaluation of SIGMA as a submucosal injection agent

**Fig. 6:**
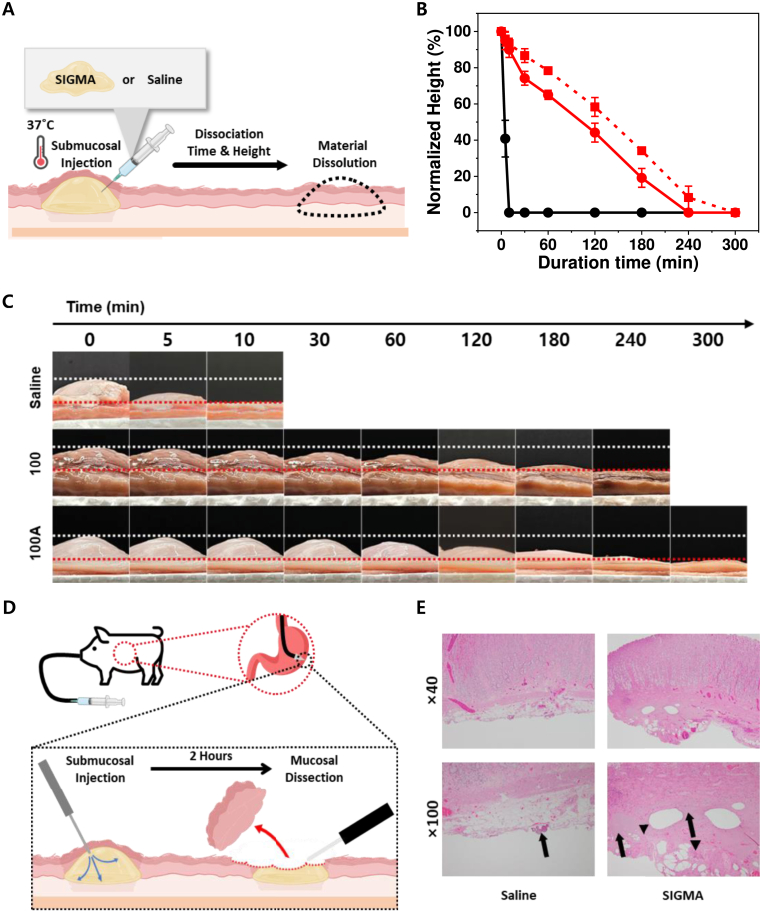
*Ex vivo* and *in vivo* performance of SIGMA as a submucosal injection agent (SIA). (A) Schematic illustration of the submucosal injection method used to assess the stability of SIGMA compared with saline under physiological temperature. (B) Retention of the elevated height of mucosal tissues after injection of saline (black), SIGMA prepared by 100 times mixing cycles without air (100, red solid line, filled dots), and with 100% air inclusion (100A, red dashed line, open dots) (n = 3, mean ± SD). (C) Representative time-course images of mucosal elevation following submucosal injection of saline (top), SIGMA without air (middle), and SIGMA with air (bottom). White dotted lines indicate the initial elevation height; red dotted lines indicate baseline. (D) Schematic illustration of the *in vivo* porcine gastric model for endoscopic submucosal dissection. (E) H&E-stained gastric tissues after submucosal injection with saline (left) or SIGMA (right), followed by submucosal dissection, showed at × 40 (top) and × 100 (bottom) magnification. Black arrows denote basophilic cells; black arrowheads indicate fibroblasts. (For interpretation of the references to color in this figure legend, the reader is referred to the Web version of this article.)

Submucosal injection agents (SIA) are clinically important because they provide a temporary fluid cushion that separates the mucosal and muscular layers. This separation prevents perforation and facilitates safe lesion removal during endoscopic mucosal resection (EMR) and related gastrointestinal procedures, where they are the most commonly employed method to create a submucosal cushion [[51], [52], [53]]. An ideal SIA must provide durable and stable mucosal elevation through simple injection, without requiring exogenous physicochemical triggers such as heat, light, or chemical crosslinkers. The properties of SIGMA align closely with these requirements. As demonstrated above, gelatin microbead fluid undergoes shear-induced gelation simply by passing through a narrow tube such as catheters or syringes, being seamlessly integrated with established endoscopic workflows. The performance of SIGMA was first compared with saline, the current clinical standard, using an *ex vivo* porcine gastric model [[53], [54], [55]]. Each material was injected into the submucosa, and mucosal elevation was monitored over time (Fig. 6A). Saline cushions collapsed within approximately 10 min, with normalized elevation height decreasing rapidly (Fig. 6B, black; Fig. 6C, top). In contrast, SIGMA produced stable and long-lasting cushions. The hydrogel was prepared using the syringe-mixing method described in Fig. 2A, employing 100 reciprocating cycles, with or without air inclusion. In the absence of air, SIGMA maintained a well-defined tissue elevation for up to 240 min (Fig. 6B, red solid; Fig. 6C, middle). Remarkably, air-mixed formulations exhibited even greater persistence, sustaining submucosal cushions for nearly 300 min (Fig. 6B, red dashed; Fig. 6C, bottom). Building on these *ex vivo* results, we next assessed the performance of SIGMA’s SIA ability *in vivo* (Fig. 6D). Both SIGMA and saline were injected into the gastric submucosa, followed by 2-h observation period. Endoscopic submucosal dissection was then performed, and the resected tissues were processed for hematoxylin and eosin (H&E) staining. Histological analysis demonstrated favorable tissue compatibility, with no evidence of inflammatory infiltration, congestion, or erosion in either the saline or SIGMA groups (Fig. 6E). In SIGMA-treated sites, mild basophilic changes (**black arrows**) and occasional fibroblasts (**black arrowheads**) were noted, but these findings were comparable to saline controls, consistent with normal wound healing. Overall, no signs of acute toxicity or adverse tissue reactions were observed, confirming that SIGMA is a safe and biocompatible submucosal injection agent for endoscopic submucosal dissection procedures. Together, these findings confirm that SIGMA is well suited for use as a submucosal injection agent. Its ability to form hydrogels solely through shear during tube passage, combined with superior mucosal lift performance and favorable histological outcomes, positions SIGMA as a promising and clinically compatible platform for EMR and related endoscopic procedures.

Currently, HA solution (e.g., MucoUp, Ksmart; typical Mw ∼500–1200 kDa) has also been widely used as SIA along with saline in clinical settings [43]. It functions primarily as pre-formed viscoelastic solutions that generate an immediate “fluid cushion” upon injection. In contrast, SIGMA is delivered as a flowable gelatin microbead slurry but is designed to assemble into a cohesive bead–bead network during confined catheter/needle flow. HA cushions are governed by solution viscoelasticity and tissue spreading, whereas SIGMA’s cohesion arises from a physically assembled network that is less prone to immediate flow-back/dispersion and is consistent with improved lift persistence in our gastric models. However, direct comparison with HA solution and SIGMA hydrogels remains a future study. In SIA practice, loss of lift often reflects tissue absorption/dispersion rather than true material degradation because solutions quickly become visually indistinguishable as they spread, defining a precise *in vivo* degradation profile for HA solutions is inherently difficult. By forming a localized particulate network, SIGMA is expected to resist rapid dispersion and exhibit a slower loss of visual detectability, with biodegradation proceeding via gelatin resorption. Finally, both HA and gelatin have established supply chains. However, SIGMA requires an added microbead fabrication step, so cost competitiveness will depend on production scale and process optimization.

## 4 Conclusion

This study presents an unprecedented gelation system using gelatin microbeads, termed SIGMA (Shear-Induced Gelation by Microbead Aggregation), that undergoes shear-induced 3D bulk gelation without exogenous physicochemical triggers. In clinical practice, shear stress is an unavoidable factor generated as injectable biomaterials pass through confined delivery devices such as syringes, catheters, or endoscopic needles. By reformulating gelatin into flowable microbeads, SIGMA transforms this unavoidable mechanical input into a constructive driving force for gel formation. Under confined flow, interfacial shear activates surface gelatin chains on the microbeads, promoting intermolecular association and bead-to-bead adhesion, ultimately yielding a cohesive and self-supporting hydrogel. The gelation kinetics and mechanical properties of SIGMA were precisely tunable by modulating flow path length, mixing cycles, particle size, and air–liquid interface exposure, yielding storage moduli up to ∼5 kPa and compressive strengths near 800 kPa. CD and GPC supported a shear-induced, physically assembled networking mechanism without chain scission, which triggered bead-to-bead network formations. Thus, SIGMA platform enabled post-encapsulation of various payloads with tunable diffusion-controlled release governed by shear history in clinical settings. *In vitro* and *in vivo* studies confirmed cytocompatibility, predictable biodegradation, systemic safety, and durable submucosal lifting performance. Collectively, these findings establish SIGMA as a reagent-free, clinically adaptable hydrogel system that redefines injection-induced shear from an engineering constraint into a practical tool for minimally invasive therapy and localized drug delivery.

## CRediT authorship contribution statement

**Yu Ri Nam:** Conceptualization, Methodology, Visualization, Writing – original draft, Writing – review & editing. **Yeongjin Lee:** Data curation, Investigation, Methodology, Visualization, Writing – original draft, Writing – review & editing. **Keumyeon Kim:** Methodology, Software. **Jeongin Seo:** Investigation, Methodology. **Jeehee Lee:** Methodology, Software. **Hee-Seung Lee:** Conceptualization, Supervision, Writing – review & editing. **Haeshin Lee:** Conceptualization, Project administration, Supervision, Writing – original draft, Writing – review & editing.

## Declaration of competing interest

The authors declare that they have no known competing financial interests or personal relationships that could have appeared to influence the work reported in this paper.

## Data Availability

Data will be made available on request.
