# Supplementary material for: SIGMA: Shear-induced gelation by microbead aggregation in tubular flow systems
Source: Mater Today Bio. 2026 Apr 13;38:103120. doi: 10.1016/j.mtbio.2026.103120 (PMC13099511; doi:10.1016/j.mtbio.2026.103120)
Supplement: Multimedia component 1 [file mmc1.docx]

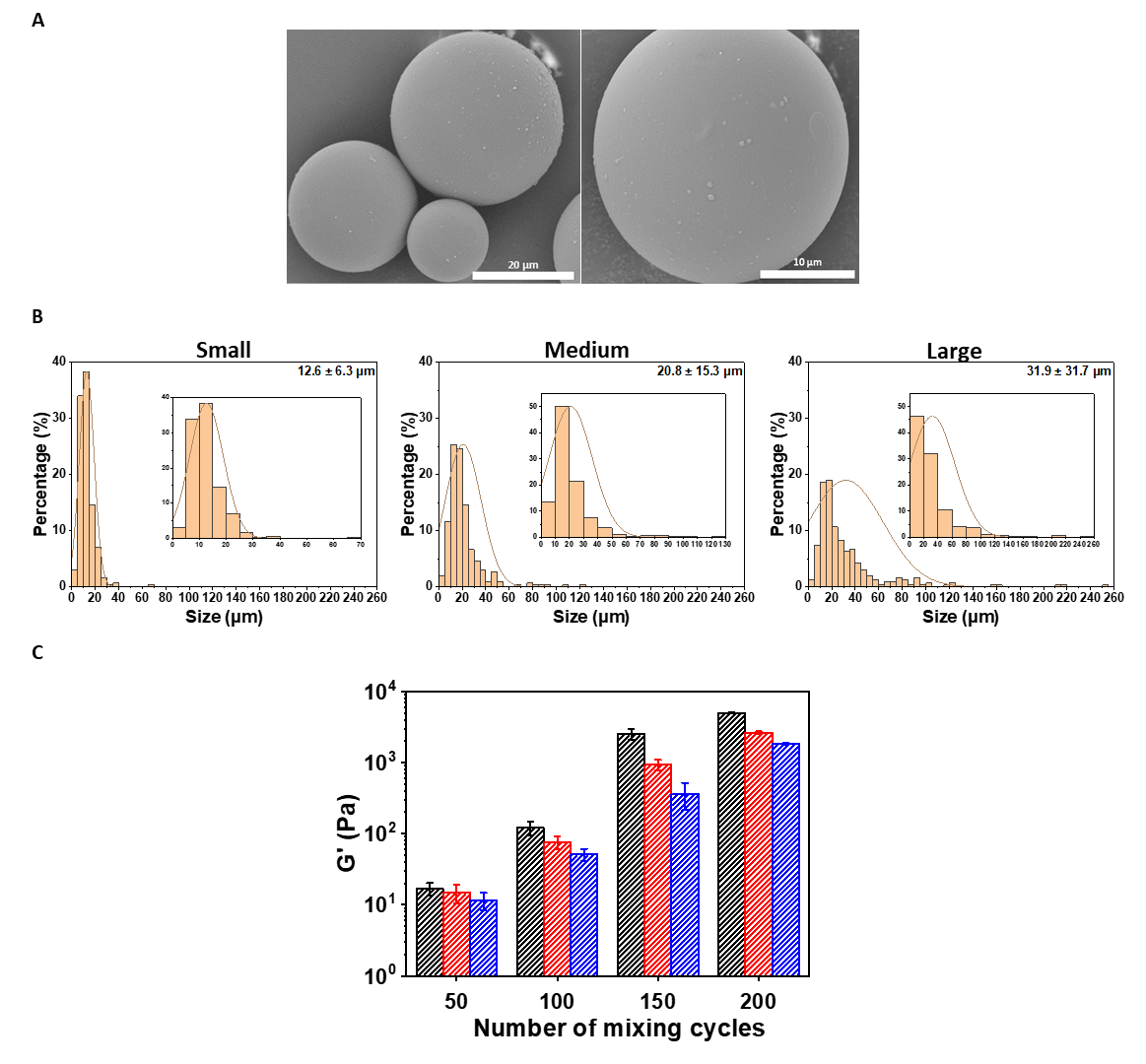


**Figure S1.** Characterization of GM and size-dependent shear-induced gelation behavior. (A) Representative SEM images of GMs, showing spherical morphology and surface characteristics. (B) Quantitative size analysis of GM classified into small, medium, and large groups (n = 300, mean ± SD). (C) Storage modulus (G') of SIGMA prepared from size-classified GMs (small, black; medium, red; large, blue) as a function of mixing cycles (n = 3, mean ± SD).


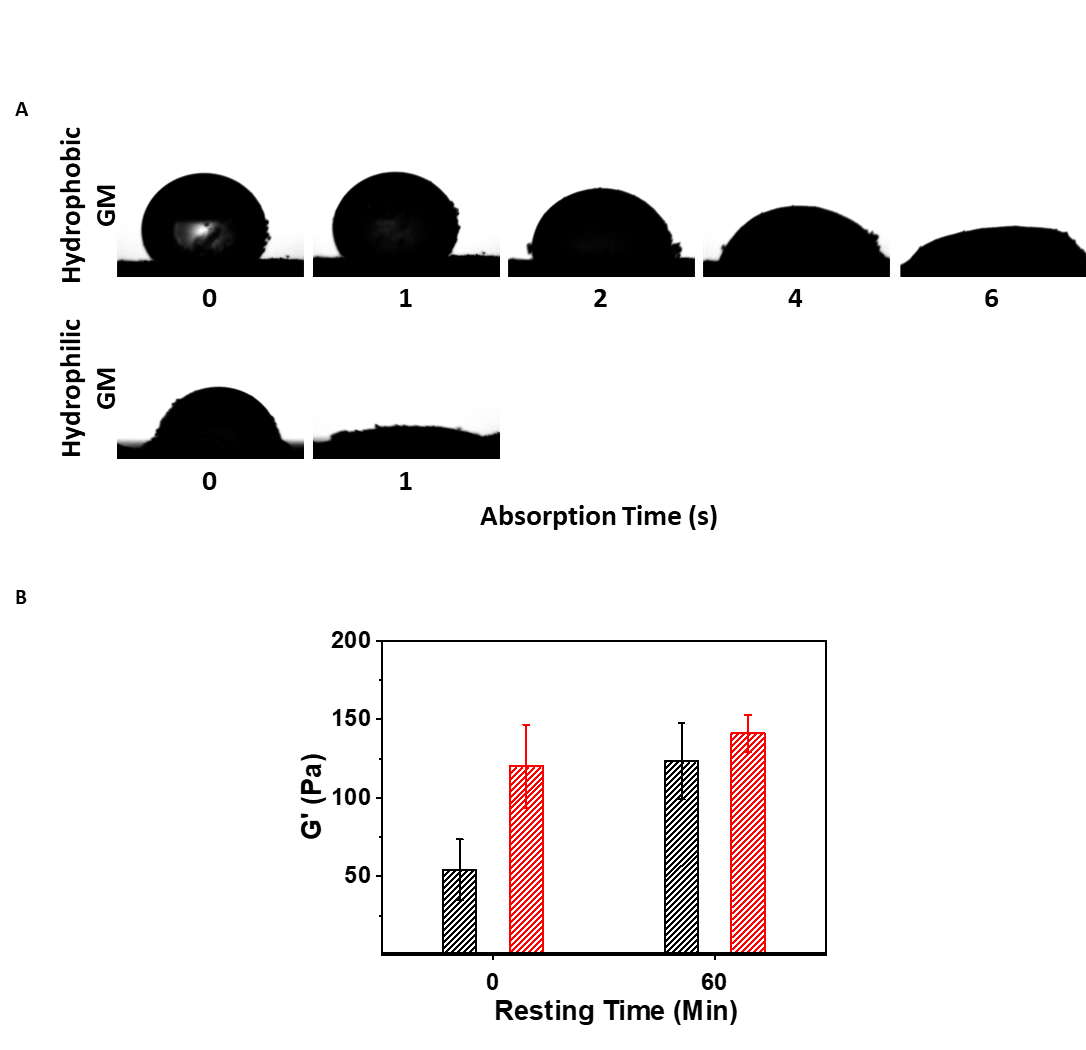


**Figure S2.** Surface wettability and hydration-dependent shear response of GM. (A) Time-resolved images acquired using a contact angle analyzer showing water absorption behavior of hydrophobic GM surface and hydrophilic GM surface. (B) Storage modulus (G') of SIGMA prepared from hydrophobic GM (black) and hydrophilic GM (red) after resting for 0 min (left) and 60 min (right) prior to measurements (n = 3, mean ± SD).


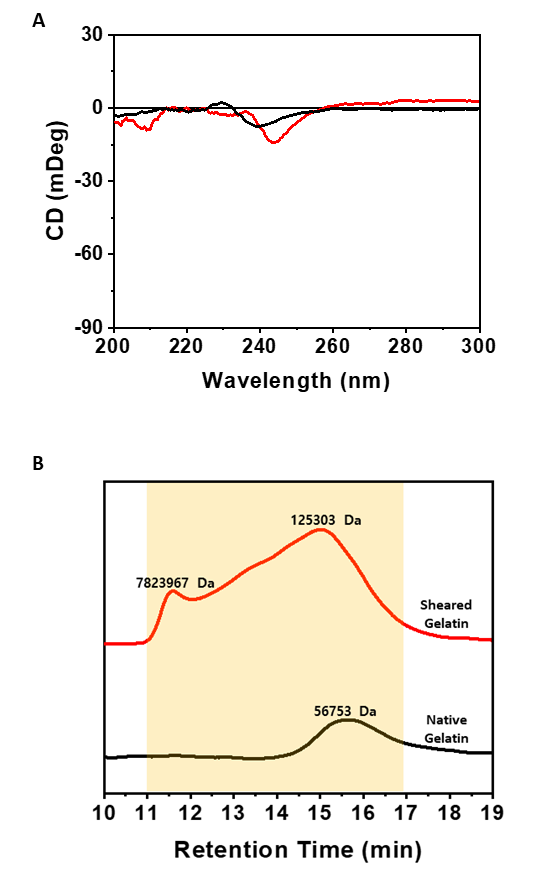


**Figure S3.** Structural and molecular analysis of native and sheared gelatin. (A) CD spectra of native gelatin (black) and sheared gelatin (red). (B) GPC chromatograms of native gelatin (black) and sheared gelatin (red).


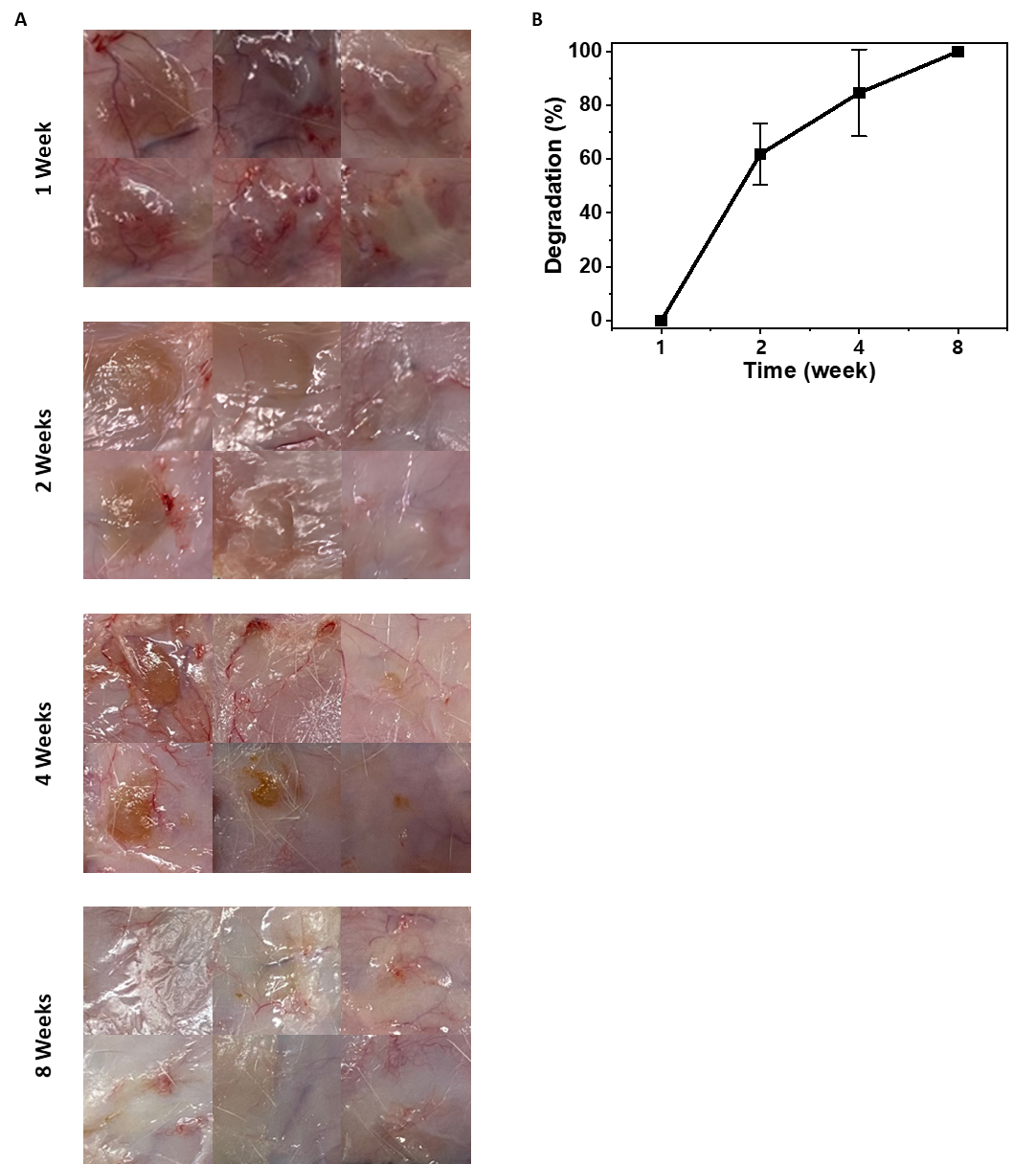


**Figure S4.** *In vivo* degradation of SIGMA following high-volume subcutaneous injection in SD rats. (A) Representative photographs showing in vivo degradation of SIGMA prepared by 100 mixing cycles after subcutaneous injection (1 mL) in SD rats at 1-, 2-, 4-, and 8-weeks post-injection. (B) Quantitative analysis of degradation area over time (n = 6, mean ± SD).


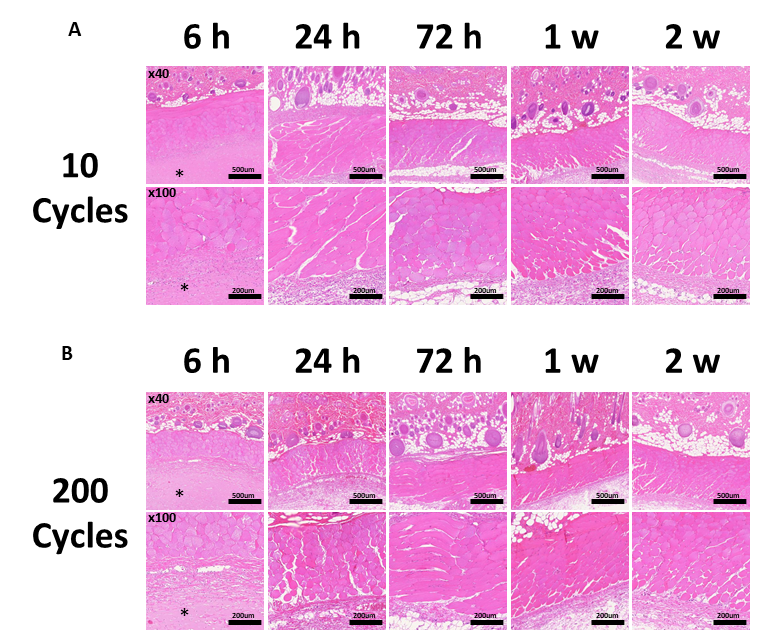


**Figure S5.** Histopathological evaluation of subcutaneous tissues following injection of SIGMA in SD rats. Representative H&E-stained histological sections of subcutaneous tissues collected at 6 h, 24 h, 72 h, 1-, and 2-weeks after subcutaneous injection (0.3 mL) of SIGMA prepared with (A) 10 or (B) 200 mixing cycles.
